# The effect of smoking on quantification of aortic stiffness by ultrasound time-harmonic elastography

**DOI:** 10.1038/s41598-022-22638-7

**Published:** 2022-10-22

**Authors:** Thomas Elgeti, Matthias Fröhlich, Kim Kathrin Wismayer, Heiko Tzschätzsch, Bernd Hamm, Ingolf Sack, Lars-Arne Schaafs

**Affiliations:** 1 Charité – Universitätsmedizin Berlin, corporate member of Freie Universität Berlin and Humboldt-Universität zu Berlin, Department of Radiology, Hindenburgdamm 30, 12203 Berlin, Germany; 2 Charité – Universitätsmedizin Berlin, Charité – Universitätsmedizin Berlin, corporate member of Freie Universität Berlin and Humboldt-Universität zu Berlin, Department of Neonatology, Augustenburger Platz 1, 13353 Berlin, Germany

**Keywords:** Diagnosis, Medical imaging

## Abstract

Smoking is a significant cardiovascular risk factor that causes stiffening of the central arteries, especially the aorta. While vessel stiffness can be determined indirectly by measuring pulse wave velocity, elastography allows image-based determination of vessel stiffness while at the same time providing information on vascular morphology. This study compares abdominal aortic wall stiffness as measured by ultrasound time-harmonic elastography (THE) in fifteen smokers and fifteen age-matched non-smoking controls without a history of cardiovascular disease. Smokers had a significantly higher abdominal aortic wall stiffness with a mean shear wave speed of 2.66 m/s (95% confidence interval (CI) 2.59–2.72 m/s) compared to 2.40 m/s (95% CI 2.34–2.47 m/s) (*p* < 0.01) in the group of non-smokers. All other baseline characteristics including aortic diameter showed no significant differences. Inter-rater variability was excellent with an intraclass correlation coefficient of 0.99 (95% CI 0.98–0.99). Our results show that THE is sensitive to subclinical stiffening of the aorta in young and middle-aged smokers even before morphological changes occur and may therefore has the potential to serve as a screening tool for early aortic abnormalities and longitudinal risk factors for cardiovascular health.

## Introduction

Smoking substantially contributes to cardiovascular mortality and even lowers life expectancy at birth^1,2^. It increases arterial stiffness and reduces the cushioning-effect of the large central arteries, especially of the aorta, which is crucial for maintaining cardiovascular health. The pulse wave ejected from the heart is to some extent absorbed by the distension of the aorta. Distension converts pulsatile energy into a rather continuous blood flow, protecting organs with low impedance capillary beds, such as the brain and kidneys, from excessive pulsatility. A physiological decrease in aortic distensibility, known as physiosclerosis, occurs with aging. Apart from lifestyle-associated factors such as smoking and hyperalimentation, arterial stiffness and altered endothelial function are influenced by diseases such as diabetes mellitus and chronic kidney disease^3–6^. For these reasons, vascular stiffness has been recognized as an independent predictor of cardiovascular health. Reliable detection of abnormal vascular stiffness is crucial for risk stratification and the planning of individual therapies^7^.

The current gold standard of arterial stiffness quantification by non-invasive methods is measurement of carotid-femoral pulse wave velocity using tonometry^8^. Several imaging techniques have been proposed to combine the measurement of arterial stiffness with the assessment of vessel morphology^9,10^. Time-harmonic ultrasound elastography (THE) has been shown to provide reproducible shear wave speed maps of the wall of the upper abdominal aorta, thus providing a direct measure of arterial stiffness in vivo while simultaneously depicting the extend of atherosclerosis or the presence of aneurysm^11,12^. While effects of aging and hypertension have already been determined by THE, there is, to the best of our knowledge, no data on the influence of smoking on aortic stiffness^12^. The aim of the present study therefore was to assess the ability of THE to measure stiffness differences in the aortic wall of adult smokers in comparison to age-matched controls*.*

## Methods

### Participants

Inclusion criteria for participants of the study group were an age of at least 18 years, active smoking and clinical absence of hyperlipidemia, diabetes mellitus and obesity with a body mass index > 30 kg/m^2^ Inclusion criteria for participants of the control group were an age of at least 18 years and absence of any cardiovascular risk factors including past or present smoking. Smokers and participants of the control group were matched for age. General exclusion criteria were pregnancy, acute myocardial infarction and stroke within 14 days before the examination. This prospective study was approved by the institutional review board and local ethics committee (Application ID: EA1/056/16) and conforms to the Declaration of Helsinki. Participants were included in the study between January 2018 and March 2019. Written informed consent was obtained from all participants. This study is registered with the WHO International Clinical Trials Registry Platform (Study ID: DRKS00013568, URL: https://www.drks.de/drks_web/setLocale_EN.do).

### Physical and hemodynamic parameters

Physical parameters (age, sex, height, and weight) were documented for each participant. Blood pressure and pulse wave velocity (PWV) were measured immediately before ultrasound elastography using an arm cuff-based, oscillometric PWV monitoring device (Agedio B900/Mobil-o-graph, I.E.M GmbH, Stolberg, Germany) while the participant sat in an upright position. All subjects were asked to refrain from strenuous physical activities for two hours before the start of the measurements.

### Experimental setup and imaging

Participants were investigated by time-harmonic elastography (THE) on a vibration-bed actuator with an integrated audio amplifier and vibration source (GAMPT mbH, Merseburg, Germany) while resting in supine position. The setup has been described in detail in other recent publications^12,13^. B-mode images using a clinical ultrasound device (Sonix MDP, Analogic, Richmond, Canada) and a 2.0-MHz phased-array transducer were obtained for visual evaluation of the aorta and to check for possible imaging artifacts. In addition, the inner diameter of the aorta was measured before THE was conducted. Time-harmonic elastography (THE) measurement was performed in the sagittal plane in the upper abdominal aorta. All participants were requested to hold their breath at an end-expiratory level in order to be able to record as much of the same section of the aorta as possible during all examinations.

As a general quality criterion, the aorta had to be visible in more than 50% of the acoustic window before elastography was started and needed to be aligned perpendicular to the acoustic beam direction. A time-harmonic signal consisting of three frequencies (60, 70 and 80 Hz) was introduced through the patient’s spine by an integrated loudspeaker to generate shear waves in the abdominal aorta. During vibration, the underlying radiofrequency data were acquired at 100 frames per second over a period of three seconds^14^. Less than three minutes were necessary to acquire B-mode images and perform THE measurements.

### Offline signal postprocessing

A separate workstation and MATLAB R2017b (MathWorks Inc.; Natick, Massachusetts, USA) was used to perform postprocessing of THE data. Stiffness maps were calculated using an algorithm developed and detailed by Tzschätzsch et al.^14^. Shear wave speed (SWS) measured in the maps in meters per second serves as a surrogate for vascular stiffness. In sum, the following postprocessing steps were undertaken: (1) for every frame, maps of tissue displacement in axial direction were generated from radiofrequency data, (2) a temporal band-pass filter and Hilbert transformation were used to decompose vibration frequencies for calculation of time- and frequency-resolved wave fields, (3) noise and motion artifacts were removed by applying a spatial band-pass filter, (4) a directional filter was applied to decompose the remaining shear wave interference pattern into plane shear waves, (5) the SWS maps for each time, frequency and direction were calculated via phase gradient, and (6) the final time-resolved stiffness map was created from the weighted average of the previously generated shear wave speed maps. Instead of earlier postprocessing approaches without temporally resolved SWS data, we here used a time-resolved map to allow measurement of SWS in the non-motion-prone diastolic phase.

Finally, SWS was extracted by placing a region of interest (ROI) on the calculated elastogram along the posterior aortic wall. All ROIs were placed by a board-certified radiologist with subspecialization in cardiovascular radiology (LAS) who had seven years of experience in vascular imaging and five years of experience in ultrasound THE and who was aware of the participants’ smoking and health status.

**Figure 1:**
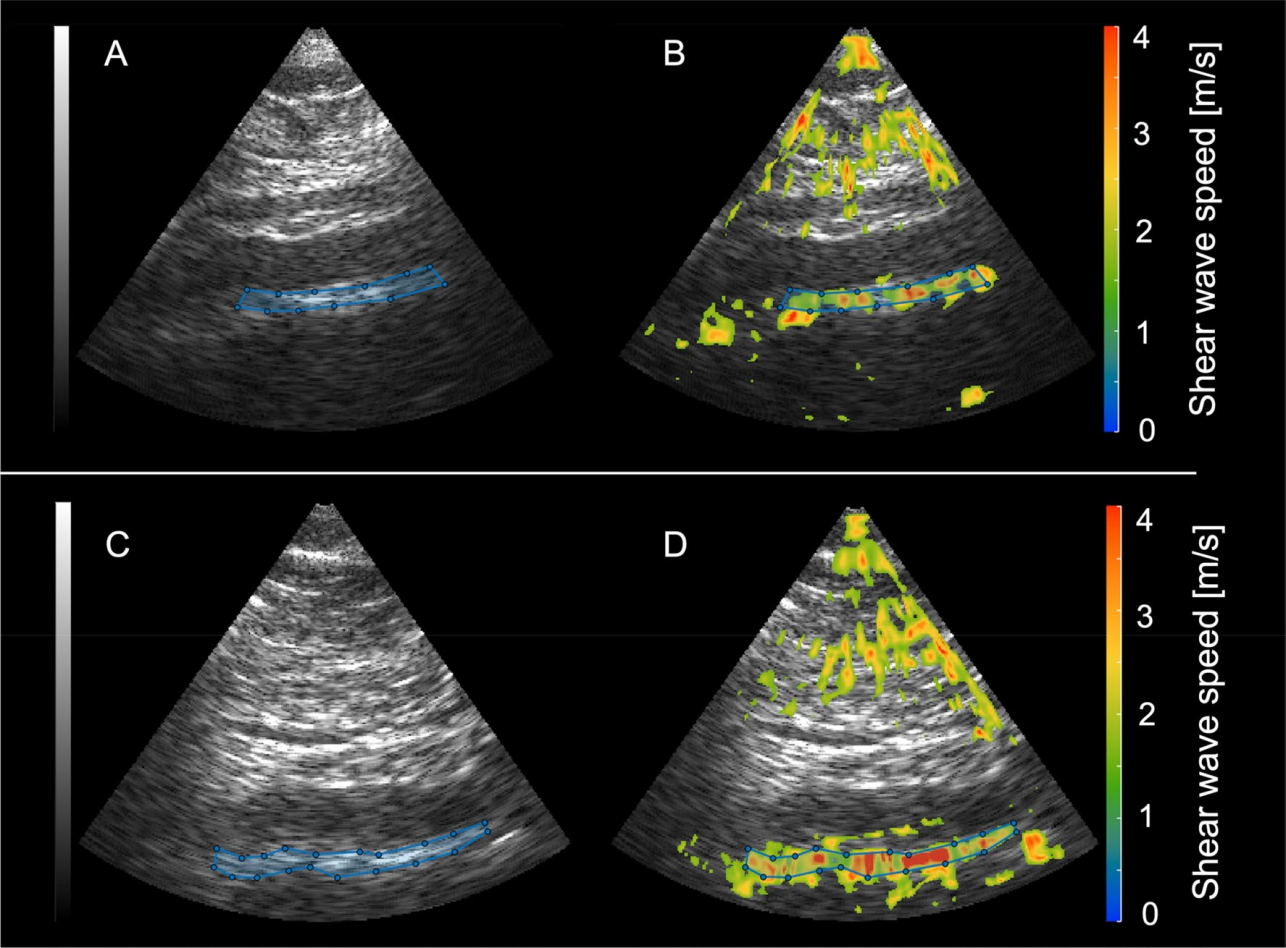
Sagittal B-mode images of the abdominal aorta and corresponding elastograms. The top row shows a sagittal B-mode image of the abdominal aorta (**A**) and the shear wave speed map (measured SWS: 2.77 m/s) (**B**) of a healthy non-smoking volunteer. The bottom row shows the corresponding B-mode image (**C**) and SWS map (measured SWS: 3.03 m/s) (**D**) of an age-matched smoker.

To determine inter-observer variability of SWS measurements, fifteen randomly chosen datasets were anonymized and re-evaluated. Re-evaluation of processed elastograms including placement ROIs along the aortic wall was performed by the initial reader and a second radiologist (TE) with fifteen years of experience in cardiovascular radiology and elastography. The second reader was blinded to the participants’ smoking and health status. Examples of SWS maps are shown in Fig. 1.

### Statistical analysis

Results are shown as medians with interquartile ranges (IQR) unless otherwise indicated. Confidence intervals (CI) are given for 95% confidence. Continuous variables were tested for normal distribution by QQ-diagrams and histograms. Variables between both groups were compared by using the nonparametric Mann–Whitney U test. Analysis of covariance (ANCOVA) was used to test the effect of smoking on aortic stiffness with age and systolic blood pressure as covariate. Sensitivity analysis was performed including aortic diameter into the model. Inter-observer variability of two raters was assessed by calculating the intraclass correlation coefficient (ICC, two-way random model). *p* values below 0.05 were considered as statistically significant. Statistical tests were performed using SPSS 27 (IBM Corp., Armonk, USA).

### Ethics declaration

This study was approved by the internal review board of Charité - Universitätsmedizin Berlin (Germany, Berlin). Application number: EA4/192/21. Written informed consent was obtained from all participants or their legal representatives. All experiments were conducted in accordance with the principles embodied in the Declaration of Helsinki and in accordance with local statutory requirements.

## Results

### Participant characteristics

A total of 30 participants were included in this study (15 in each group) with 2 female non-smokers and 5 female smokers. All demographic data and results for body measurement and hemodynamics (including PWV, blood pressure and aortic diameter) are given in Table 1. There was no normal distribution, and median and IQR are displayed. Analysis of aortic diameters revealed no significant differences between smokers (15.8 mm (95% CI 14.5–17.6 mm)) and non-smokers (16.0 mm (95% CI 14.7–19.1 mm)). None of the participants had a history of hypertension, diabetes mellitus, or hyperlipidemia.

**Table 1:** Medians and IQRs of demographic data and body measurements as well as blood pressure (BP) and aortic diameter.

Parameter	Non-smoker	Smoker	*p*-value*
Age [y]	40 (27–61)	40 (27–61)	1
BMI [kg/m^2^]	23.7 (20.7–23.9)	23.2 (21.3–25.1)	0.713
Pack years	–	10 (5.5–17.5)	
Pulse wave velocity [m/s]	5.7 (5.1–8.0)	6.2 (5.6–8.7)	0.345
Systolic BP [mm Hg]	123 (115–131)	131 (116–145)	0.486
Diastolic BP [mmHg]	76 (75–91)	79 (73–84)	0.683
Mean art. pressure [mmHg]	91 (88–105)	96 (87–110)	1
Aortic diameter [mm]	15.8 (14.5–17.6)	16.0 (14.7–19.1)	0.775

*Mann–Whitney U test.

### THE

**Figure 2:**
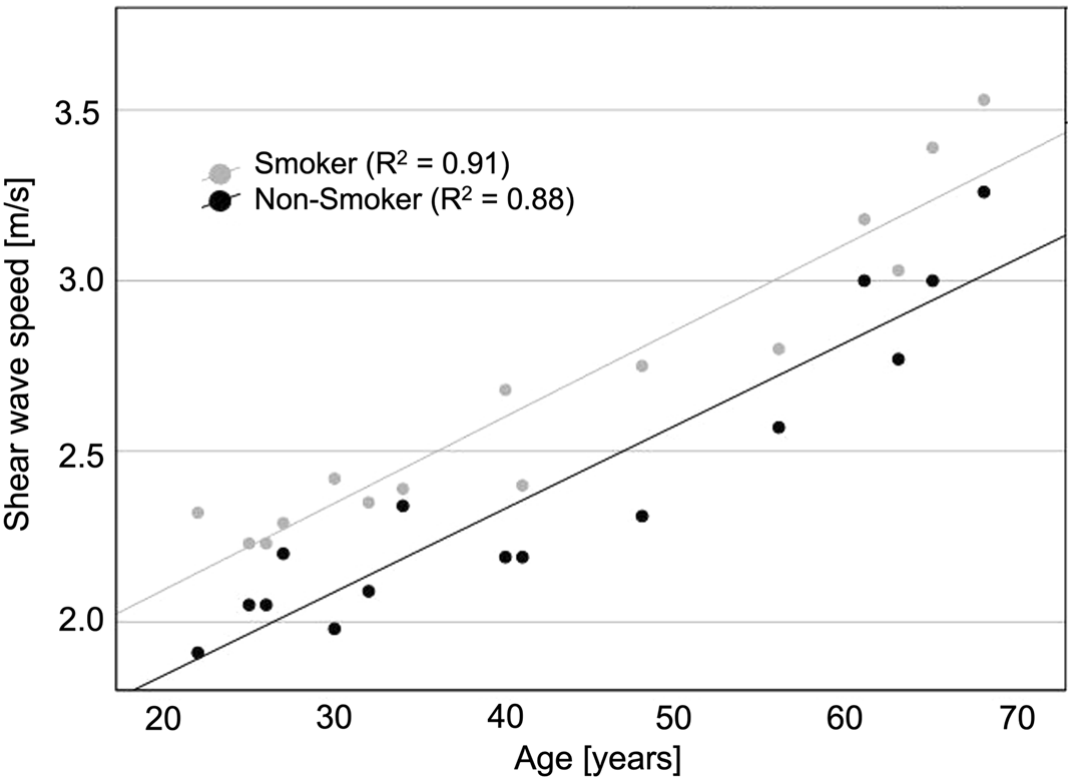
Scatter plot of shear wave speed versus age in smokers and non-smokers. Smokers (light gray dots) show a higher SWS (in meters per second) compared to age matched non-smokers (black dots).

In ANCOVA, smokers had a significantly higher mean SWS of the aortic wall of 2.67 m/s (95% CI 2.59–2.72 m/s) compared with 2.40 m/s (95% CI 2.34–2.47 m/s) in non-smokers (*p* < 0.001). These results were confirmed by sensitivity analysis including aortic diameter. Figure 2 displays the scatter plot of SWS vs. age of the study participants. Inter-rater agreement in the subgroup analysis was excellent with an ICC of 0.99 (95% CI 0.98–0.99).

## Discussion

To the best of our knowledge, this study shows for the first time that in vivo THE of the human aorta can detect significantly higher stiffness of the aortic wall (i.e., 12%) in smokers compared to non-smokers. These results were obtained in a small population of volunteers without any clinical signs of cardiovascular morbidity. THE thus shows potential to be used as an additional imaging modality for investigating and understanding end-organ pathophysiology and biomechanics of large vessels. Furthermore, THE or other elastographic techniques might provide interesting additional information on the role of smoking- and non-smoking-related vascular pathology such as rupture in the formation of aortic aneurysms. In our volunteers, THE detected stiffness changes of the aortic wall in smokers, although the aortic diameter, currently used for assessment of vascular health, showed no significant differences between the two groups^15,16^. THE has the potential advantage of providing both morphological and biomechanical information in a single examination.

Sonography has been shown to measure abdominal aortic diameter in a reproducible fashion, which is why this measure is used for screening and follow-up in current clinical practice^16,17^. A special ultrasound technique, 3D-speckle tracking, analyzes aortic wall geometry over the cardiac cycle and calculates wall stress by finite element analysis^18^. It is known that the largest aortic diameter is a strong predictor of the risk of rupture in patients with abdominal aortic aneurysm, and cut-offs for rupture risk versus the risk of surgical intervention have been published^19^. It would be desirable, however, to have a quantitative stiffness-based imaging parameter that reliably reflects potential changes of vessel wall properties in smoking and after cessation of smoking^20,21^. Evidence exist, that ultrasonographic elastography methods provide additional information on pure morphological information that can be helpful after endovascular repair of aortic aneurysms or in multiparametric vascular ultrasound imaging^22,23^. Furthermore, in the last years, 4D-flow and MR elastography have been used to investigate the complex interaction of aortic wall and diameter changes in patients with aneurysm^24–26^. In a recent study, Dong et al. found lower abdominal aortic stiffness and stiffness ratio measured by MR elastography to be associated with aneurysmal events^27^.

In our study, the diameter of the abdominal aorta was not different between smokers and non-smoking age-matched controls. SWS, however, was significantly different even when adjusting for age. This finding indicates that THE is sensitive to early structural changes of the aortic wall prior to morphological dilatation of the vessel. Due to its non-invasive nature, THE offers an excellent screening tool for longitudinal effects of smoking and other noxious substances on large vessel wall properties^28^.

Despite these promising results, our study has some limitations. First, statistical significance is shown despite the relatively small number of cases. An investigation in larger populations would yield more precise data. In addition, it would be possible to correct for other factors such as sex. The fact that one reader was not blinded to smoking status may have introduced a possible bias, but it was shown that excellent inter-rater agreement was achieved with the blinded reader. Second, THE estimates effective aortic stiffness only, which reflects elastic properties of the vessel wall and surrounding tissue and in part the prevailing arterial blood pressure at the time of measurement^29–31^. Additionally, with current technology THE does not allow determination of SWS throughout the cardiac cycle because the traveling pulse wave causes deflection of the aortic wall during systole precluding determination of systolic SWS within the vessel wall. Furthermore, stiffness measurement alone cannot differentiate between what is known as physiosclerosis, which is a process of the tunica media, and conventional atherosclerosis, which is mostly limited to the tunica intima. However, in contrast to the established, non-imaging-based methods for measuring aortic stiffness, THE offers provides both stiffness and morphological information in the B-mode image, additionally allowing detection of atherosclerotic plaque or aneurysm. Lastly, we did not consider sex-specific differences. Such differences in aortic elasticity exist, and other studies have already shown that these differences increase especially because of changes that occur in women after menopause^32^. Due to the lower median age of the volunteers in our study, the effect of sex should be marginal.

## Conclusion/summary

In summary, we have shown that, in our small group of volunteers, THE identifies an about 12% stiffer abdominal aorta in young and middle-aged smokers compared to non-smokers. These changes are detectable before morphological changes occur and may therefore serve to screen for longitudinal effects of smoking on vascular health.

## Data Availability

The datasets used and/or analyzed during the current study are available from the corresponding author on reasonable request.
